# Place of Death of People With Cancer in 12 Latin American Countries: A Population Study Using National Death Registers

**DOI:** 10.1002/cam4.70996

**Published:** 2025-06-18

**Authors:** Alisa Dannenberg, Joachim Cohen, Luc Deliens, Katja Seitz, Alicia Krikorian, Luis Fernando Rodrigues, Verónica Kramer, Alejandra Sosa Basaistegui, Alberto Alonso Babarro, Andrea Cartin, Celina Castaneda, Lesly Peralta, Cesar Villacres, Sofía Bunge, Tania Pastrana

**Affiliations:** ^1^ Department of Palliative Medicine Uniklinik RWTH Aachen Aachen Germany; ^2^ End‐of‐Life Care Research Group Vrije Universiteit Brussel (VUB) and Ghent University Brussels Belgium; ^3^ Group of Pain and Palliative Care School of Health Sciences, Universidad Pontificia Bolivariana Medellín Colombia; ^4^ Palliative Care Unit Barreto's Cancer Hospital Barretos Brazil; ^5^ National Cancer Institute Santiago de Chile Chile; ^6^ National Cancer Institute Montevideo Uruguay; ^7^ Unidad de Cuidados Paliativos Hospital Universitario La Paz Madrid Spain; ^8^ Centro de Cáncer y Hematología Hospital Metropolitano Universidad de Costa Rica San José Costa Rica; ^9^ Facultad de Medicina Universidad Autonoma del Estado de Mexico Ciudad de México Mexico; ^10^ Department of Palliative Care and Pain National Cancer Institute Capiata Paraguay; ^11^ Servicio de Geriatría, Hospital Alberto Sabogal Sologuren – Essalud Callao Peru; ^12^ National Cancer Institute Buenos Aires Argentina

**Keywords:** administrative data, cancer, Latin America, place of death

## Abstract

**Background:**

Cancer is one of the leading causes of death in Latin America. This study aims to compare the percentage of home and hospital deaths among cancer patients in 12 Latin American countries and to examine associated factors.

**Methods:**

We carried out a population‐level observational study using death certificate data from Argentina, Brazil, Chile, Colombia, Costa Rica, Ecuador, El Salvador, Guatemala, Mexico, Paraguay, Peru and Uruguay. Data from cancer decedents (ICD‐10 C00–C97) for a full year (January 1 to December 31) with available data (2016–2018) were included. We used descriptive statistics and multivariable binary logistic regression analysis in each country and between countries to describe the distribution in place of death and examine associated sociodemographic, clinical and ecological factors.

**Results:**

Cancer was the underlying cause of 491,929 deaths. An average of 31.1% occurred at home, from a range of 14.9% in Brazil to 81% in Guatemala. The variation remains after controlling for sociodemographic factors and cancer types. Patients who are older, live in rural areas, have lower educational levels, and a solid cancer history are more likely to die at home. Countries with more hospital beds and physicians, better Universal Health Coverage, higher Human Development Index, and higher health expenditure per capita had fewer home deaths.

**Conclusion:**

Factors affecting place of death patterns in Latin America are country‐specific and the results can only partially be explained by sociodemographic, clinical and ecological factors. Our results may be used to improve palliative care according to the current country‐specific distribution of place of death.

## Graphical abstract

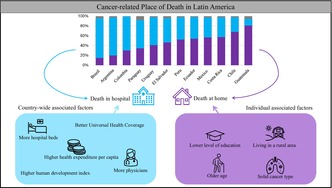

## 1 Introduction

Place of death (PoD) is often regarded as an important quality metric of end‐of‐life care [1]. Previous studies have focused mainly on high‐income countries, where hospitals, hospices and home care options tend to be more accessible and available at the end of life. PoD in Latin America, a region encompassing a range of lower middle‐income to high‐income countries, has been relatively understudied. There have been significant improvements in health services and universal health coverage over the past two decades, but disparities between and within countries persist. Cancer is one of the leading causes of death in Latin America and the leading cause of premature death in eight countries across the region. Epidemiological shifts in the next decade will trigger increased numbers of cancer cases and deaths [2].

Studies on preferred PoD for people with cancer in Latin American countries report similar findings to those in European or North American countries: 65% of patients with advanced cancer and their caregivers in Chile reported a preference for home death [3], as did 77.4% of patients with advanced cancer in Brazil in the scenario of receiving home‐based visits as needed [4]. However, the reported percentage shrinks to 17.4% in the scenario of severe and uncomfortable symptoms, indicating that the circumstances of death and care may influence these preferences.

Evidence of actual PoD, however, reveals heterogeneity [5, 6]. Studies from Latin America, including Brazil [7, 8], Colombia [9], Chile [10, 11] and Mexico [12, 13, 14, 15] reported home deaths in 17.7% (Brazil) to 57.7% (Mexico) of cancer patients and hospital death in 30.1% (Chile) to 88.2% (Brazil). A comparative regional study reported that cancer as an underlying cause of death was associated with home (as opposed to hospital) death in most of the countries [16]. The international literature associates the PoD among cancer patients with sociodemographic (age [7, 8, 10, 12, 13, 15, 16, 17], level of education [7, 8, 12, 13, 15, 16], level of urbanization [12, 15, 16, 17], gender [7, 8, 15, 16, 17], race [7, 8] and marital status [8, 16]), illness‐related (cancer type [7, 8, 12, 13, 15]) and health system‐related factors (density of hospital beds [7, 12, 13, 17], Human Development Index [8]).

Rational planning for effective end of life service integration into the existing health systems [1] entails understanding variations between PoD among cancer patients in Latin America. This study aims to describe where cancer patients die in 12 Latin American countries, and which factors are associated with cancer‐related death at home or in the hospital. The research questions are: What proportion of cancer‐related deaths occur at home or in the hospital? Which factors are associated with death at home or in the hospital? How can cross‐country differences be explained?

## 2 Method

### 2.1 Design

We collected and integrated total population mortality databases from Argentina, Brazil, Chile, Colombia, Costa Rica, Ecuador, El Salvador, Guatemala, Mexico, Paraguay, Peru and Uruguay, and performed a comparative analysis of the place of death.

We selected all decedents with cancer as the primary cause of death, identified by codes C00–C97 of the International Classification of Diseases 10th revision (ICD‐10) from the dataset collected in 2019 by the project ‘Place of Death in Latin America’ [16]. The dataset comprises mortality statistic records of a 1‐year period, using the most recent year available (2016, 2017 or 2018) at the time of data collection. It includes the variables PoD, cause of death (coded as ICD‐10 codes) and sociodemographic factors. The quality of the data was assessed to be feasible for international comparison [18].

### 2.2 Measures

The dependent variable PoD was categorized as occurring ‘at home’, ‘in hospital’ or ‘elsewhere’. In every country, ‘home death’ was recorded on the death certificate, while the category ‘hospital’ was either available as one category (five countries) or was recoded from similar categories with the support of the local partner [18]. The categories put together as ‘elsewhere’ differed between the countries, including ‘public place’, ‘workplace’, ‘other health care facility’, ‘nursing home’, ‘ambulance’, ‘prison’, ‘in transit’ and ‘other place’.

The independent variables include sociodemographic factors. Information on sex and age was available for each country. Information on marital status was missing for Argentina, and level of education was missing for Costa Rica and El Salvador. For the remaining countries, level of education was categorized into five categories: ‘less than primary complete’, ‘primary complete’, ‘secondary I complete’, ‘secondary II complete’ and ‘tertiary complete’ [16]. Place of residence was reported as rural or urban with no data available for Argentina, Guatemala, Paraguay and Peru.

Cancer was differentiated into solid (C00–C80, C97) and hematologic (C81–C96) types as an illness‐related factor, based on previous findings showing a higher likelihood of hospital death for people with hematologic cancer [6, 7, 12, 13, 19, 20].

Covariates representing ecological (i.e., country‐level) health system and socio‐economic characteristics were included. Data for hospital beds, physicians and nurses and midwives per 10,000 inhabitants, healthcare expenditure per capita, gross national income per capita, and Gini Index were sourced from the World Bank [21]. The Human Development Index data was obtained from the United Nations [22] and data on Universal Health Coverage was taken from the World Health Organization (WHO) [23]. The data refer to the year of the death certificate collected for each country, the closest year available, or the median of the previous and following year. Information on palliative care resources per 1 million inhabitants, distribution of opioids, and information on existing PC was extracted from the literature [24].

### 2.3 Statistical Analysis

We described the percentage of home and hospital deaths as well as the individual characteristics of the decedents. Since we used population‐level data, no inferential statistics were calculated.

We conducted a multivariable binary logistic regression analysis with home death versus hospital death as the dependent variable to examine the association between PoD and the independent variables age, sex, marital status, type of cancer (hematologic vs. solid), level of education, rural or urban residence for each country individually. For all countries involved PoD labeled as ‘elsewhere’ (3.3%) and ‘missing’ (1.3%) were excluded in this regression model for comparability between the two primary locations: ‘home’ and ‘hospital’. ‘Elsewhere’ contained various places with only a small number each, differing between countries. Furthermore, it could not be logically grouped with either ‘home’ or ‘hospital’ without changing the goal of the analysis. However, we conducted a sensitivity analysis with two additional binary logistic regression models, changing the outcome variable PoD to ‘home versus elsewhere’ and ‘hospital versus elsewhere’ to verify our findings. Variables without data for some countries were not included in the regression for those countries. Additionally, one person each in Costa Rica and Mexico were excluded due to the missing label for age and sex, respectively. This led to 469,208 valid cases included in the regression. Variables were checked for multicollinearity with variable inflation factor (VIF) and tolerance tests, and no variable had to be excluded due to collinearity.

To examine whether the country differences in the likelihood of cancer‐related death at home can be explained by the factors included in the model, we conducted a separate binary logistic regression with the dependent variable PoD (‘home’ vs. ‘hospital’). PoD defined as ‘elsewhere’ or missing values were again excluded, leading to 469,210 deaths included. We chose a hierarchical method and entered variables stepwise. First, only the independent variable ‘country’ was included (Model 1), then we added ‘type of cancer (solid vs. hematologic)’ (Model 2) and additionally ‘age’, ‘sex’, ‘level of education’ and ‘civil status’ in a backwards selection (Model 3). Area of residence was left out due to differences in categorization between the countries. We chose Uruguay as the country of reference because it was reported to be the country with the best palliative care development in the region [24].

To measure the association of health care‐related factors, the Pearson's correlation coefficient between the percentage of home death and ecological factors was calculated. Percentages of home death were hereby adjusted to age (see Supporting Information). A Pearson correlation of < 0.3 was considered negligible, 0.3–0.5 low, 0.5–0.7 medium and > 0.7 strong.

All analysis was performed with IBM SPSS Statistics V.29.

### 2.4 Ethics

The project was approved by the Ethics committee of RWTH Aachen University (EK 206/19).

## 3 Results

Cancer was reported as the cause of 491,929 deaths, ranging from 3161 in El Salvador to 217,697 in Brazil. 1808 (57.2%) cancer deaths in El Salvador were in women, a rate that decreased to 3591 (45.5%) deaths in Uruguay, but that remained above 50% in half of the included countries (Colombia, Ecuador, El Salvador, Guatemala, Mexico and Peru). More than half of cancer deaths (53.2%) were premature deaths, occurring below 70 years of age, with the highest percentage in Guatemala (59.5%) and the lowest in Uruguay (39.8%) (Table 1).

**TABLE 1:** Death from cancer and sociodemographic characteristics in 12 Latin American countries (*N* = 491,929).

Country	Total population [21]	Cancer deaths	Cancer type	Sex	Age				Residence	Marital status					Level of education^a^				
			Hematologic (%)	Female (%)	0–59 (%)	60–69 (%)	70–79 (%)	> 80 (%)	Rural (%)	Single (%)	Married (%)	Widowed (%)	Divorced (%)	Stable relationship (%)	I (%)	II (%)	III (%)	IV (%)	V (%)
Argentina (2017)	44,044,811	62,731	6.8	48.3	23.2	25.1	27.7	24.0	NA	NA	NA	NA	NA	NA	18.8	51.8	19.1	2.2	8.1
Brazil (2017)	208,504,960	217,697	7.0	47.4	30.3	25.2	24.3	20.2	13.5	23.9	44.3	20.4	8.1	3.3	43.9	25.1	17.5	4.8	8.7
Chile (2016)	18,038,879	26,027	7.8	47.5	20.4	22.3	28.2	29.1	12.4	23.7	65.2	8.7	2.4	No option	46.8	19.1	NA	27.8	6.3
Colombia (2017)	48,351,671	42,610	9.9	50.9	29.3	22.9	25.0	22.8	10.1	19.8	38.0	21.6	5.8	14.8	22.1	44.2	12.8	9.1	11.8
Costa Rica (2016)	4,945,205	4857	10.8	46.7	25.8	22.2	24.4	27.6	21.5	17.9	48.5	19.6	8.3	5.8	NA	NA	NA	NA	NA
Ecuador (2017)	16,696,944	11,016	11.7	50.9	29.2	19.6	24.0	27.2	23.0	29.6	46.3	15.3	6.0	2.8	15.2	52.5	13.3	8.2	10.7
El Salvador (2017)	6,266,654	3161	10.4	57.2	35.8	21.0	22.6	20.6	28.7	44.0	44.6	6.4	3.0	2.0	NA	NA	NA	NA	NA
Guatemala (2017)	16,087,418	7738	9.6	54.9	38.1	21.4	21.9	18.6	NA	51.4	47.7	Not reported^b^		1.0	45.7	34.6	4.7	10.7	4.3
Mexico (2017)	122,839,258	84,142	11.1	51.2	34.6	22.4	23.7	19.3	18.8	17.8	50.1	19.5	4.5	8.1	37.4	25.3	15.0	9.6	12.6
Paraguay (2017)	6,355,404	4329	8.8	47.1	34.5	23.6	23.3	18.7	NA	38.9	46.2	10.4	1.5	2.9	35.8	37.7	NA	18.1	8.4
Peru (2017)	31,605,486	19,737	10.4	53.6	28.4	20.2	25.6	25.8	NA	35.5	48.3	10.0	2.0	4.4	34.9	25.9	NA	25.3	13.9
Uruguay (2018)	3,427,042	7884	9.0	45.5	17.4	22.4	28.6	31.6	0.4	15.8	45.1	27.1	12.0	No option	14.7	52.1	13.3	10.9	9.0
Total		491,929	8.3	48.9	29.4	23.8	24.9	21.8	11.8	20.5	40.4	16.1	5.5	4.4	37.2	28.6	13.9	8.7	9.6

*Note:* Percentages are percentages of all cancer deaths, except where specified otherwise. NA, not available; missing are left out. For table including missing values see Supporting Information. High amounts of missing values in Uruguay on place of residence (68.3%) and level of education (71.2%); and in Argentina on level of education (67.3%).

^a^ Level of education: I = less than primary complete; II = primary completed; III = secondary I completed; IV = secondary II completed; V = tertiary completed.

^b^ In Guatemala only single, married and stable relationships are options for marital status.

### 3.1 Place of Death

**FIGURE 1:**
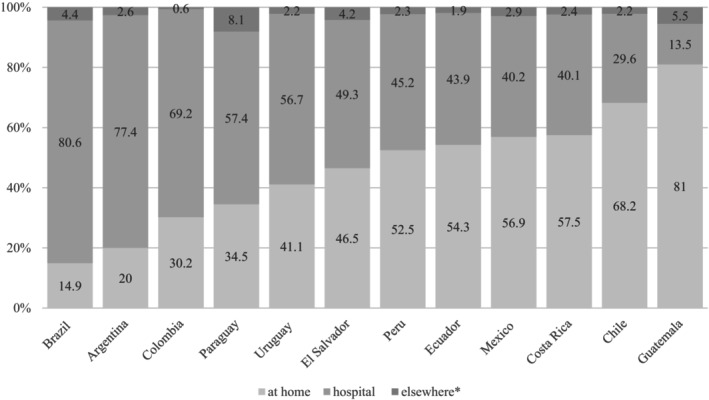
Percentages of place of death for each country. Percentage of unknown: AR = 1.1%, CR = 0.2%, GT = 0.8%, MX = 1.0%, PR = 24% (left out). *Includes: Public place (BR, CO, CR, SV, GT, MX, PY, PE, UY), workplace (CO, GT, PE, UY), other health care facility (BR, CO, CR, EC, SV, GT, UY), nursing home (CR), ambulance (CR), prison (UY), in transit (PE), other place (all countries). Missing values were not included.

A regional average of just under one‐third of all cancer deaths, 31.3%, occurred at home, ranging from 14.9% in Brazil to 81% in Guatemala (Figure 1). The remaining two‐thirds, 65.3%, occurred in the hospital, ranging from 13.5% in Guatemala to 80.6% in Brazil. Information on PoD was not available for 6424 cases (1.3%), with an especially high amount in Peru (24%).

### 3.2 Factors Associated With Place of Death (Per Country Analysis)

The likelihood of dying at home increased with age in all countries (Table 2). Compared to those younger than 60 years, the odds ratio (OR) of cancer‐related death at home rather than in hospital for those aged 60–69 in seven countries ranged from 1.15 (95% CI = 1.08–1.22) in Colombia to 1.54 (95% CI = 1.24–1.9) in El Salvador. The two older age groups showed a significantly higher likelihood of cancer‐related death at home rather than in hospital compared to those younger than 60 in all countries. For those decedents aged 70–79, the OR ranged from 1.16 (95% CI = 1.05–1.28) in Peru to 1.8 (95% CI = 1.46–2.21) in El Salvador, and for those deceased aged 80 and older, the OR ranged from 1.3 (95% CI = 1.18–1.45) in Peru to 3.32 (95% CI = 2.66–4.14) in El Salvador.

**TABLE 2:** Multivariable binary logistic regression per country: Associations with home death versus hospital death; odds ratio and 95% confidence interval given.

Country	AR	BR	CL	CO	CR	EC	SV	GT	MX	PY	PE	UY
Number included	60,402	207,977	25,444	42,335	4733	10,812	3028	7249	80,883	3979	14,656	7709
Sex (Ref: Male)												
Female	1.03 (0.99–1.07)	**0.84** (**0.82–0.86**)	**1.13** (**1.07–1.19**)	0.99 (0.94–1.22)	0.95 (0.84–1.07)	1.06 (0.97–1.15)	1.12 (0.95–1.31)	1.04 (0.90–1.20)	**1.06** (**1.03–1.09**)	**0.81** (**0.71–0.93**)	0.97 (0.9–1.04)	**1.18** (**1.07–1.31**)
Age (Ref: 0–59)												
60–69	**1.25** (**1.17–1.33**)	**1.19** (**1.15–1.24**)	**1.17** (**1.08–1.27**)	**1.15** (**1.08–1.22**)	1.06 (0.9–1.26)	1.10 (0.98–1.23)	**1.54** (**1.24–1.9**)	1.18 (0.98–1.41)	**1.29** (**1.24–1.34**)	1.13 (0.94–1.36)	0.96 (0.87–1.06)	**1.22** (**1.04–1.43**)
70–79	**1.66** (**1.57–1.77**)	**1.53** (**1.48–1.59**)	**1.41** (**1.31–1.53**)	**1.34** (**1.26–1.42**)	**1.18** (**1–1.4**)	**1.18** (**1.05–1.32**)	**1.8** (**1.46–2.21**)	**1.53** (**1.26–1.86**)	**1.68** (**1.61–1.75**)	**1.25** (**1.04–1.51**)	**1.16** (**1.05–1.28**)	**1.65** (**1.41–1.92**)
80 and older	**2.53** (**2.38–2.69**)	**2.3** (**2.21–2.39**)	**1.95** (**1.8–2.12**)	**1.87** (**1.75–2.00**)	**1.78** (**1.49–2.13**)	**1.67** (**1.48–1.88**)	**3.32** (**2.66–4.14**)	**2.53** (**1.98–3.24**)	**2.61** (**2.49–2.75**)	**1.74** (**1.42–2.13**)	**1.30** (**1.18–1.45**)	**2.28** (**1.94–2.67**)
Residence (Ref: Urban)												
Rural	NA	**2.38** (**2.31–2.45**)	0.96 (0.89–1.05)	**1.10** (**1.03–1.18**)	1.02 (0.88–1.18)	**1.32** (**1.2–1.45**)	**3.11** (**2.61–3.7**)	NA	**2.08** (**1.99–2.17**)	NA	NA	0.69 (0.44–1.08)
Marital status (Ref: Single)												
Married	NA	**0.87** (**0.84–0.89**)	**1.19** (**1.12–1.27**)	0.98 (0.92–1.05)	**1.30** (**1.10–1.54**)	**1.1** (**1–1.212**)	0.93 (0.79–1.09)	**1.17** (**1.02–1.35**)	**0.9** (**0.86–0.94**)	0.96 (0.83–1.12)	**1.1** (**1.01–1.19**)	1.15 (0.99–1.34)
Widowed	NA	**0.89** (**0.85–0.92**)	**1.15** (**1.03–1.29**)	0.99 (0.92–1.06)	**1.27** (**1.03–1.57**)	**1.11** (**1–1.31**)	0.89 (0.63–1.24)	NA	**0.88** (**0.83–0.93**)	1.09 (0.86–1.39)	**1.21** (**1.06–1.38**)	1.12 (0.94–1.34)
Divorced	NA	**0.82** (**0.77–0.87**)	1.10 (0.92–1.32)	0.93 (0.84–1.04)	1.14 (0.89–1.46)	0.97 (0.80–1.15)	1.08 (0.69–1.70)	NA	**0.83** (**0.77–0.9**)	1.48 (0.87–2.52)	1.25 (0.98–1.6)	0.89 (0.73–1.09)
Stable relationship	NA	**1.23** (**1.15–1.32**)	NA	**0.85** (**0.79–0.92**)	**0.67** (**0.50–0.89**)	**1.41** (**1.10–1.81**)	1.28 (0.71–2.28)	2.42 (0.86–6.83)	**1.08** (**1.01–1.15**)	**1.78** (**1.21–2.61**)	**2.39** (**1.98–2.89**)	NA
Cancer type (Ref: Solid)												
Hematologic	**0.44** (**0.39–0.49**)	**0.39** (**0.36–0.41**)	**0.34** (**0.31–0.38**)	**0.35** (**0.31–0.38**)	**0.41** (**0.34–0.49**)	**0.30** (**0.26–0.34**)	**0.26** (**0.19–0.35**)	**0.24** (**0.2–0.29**)	**0.33** (**0.31–0.35**)	**0.45** (**0.34–0.61**)	**0.35** (**0.31–0.39**)	**0.52** (**0.44–0.63**)
Level of education (Ref: Less than primary)												
Primary complete	0.97 (0.90–1.06)	**0.77** (**0.75–0.8**)	0.99 (0.92–1.07)	**0.85** (**0.80–0.90**)	NA	**0.70** (**0.62–0.80**)	NA	**0.62** (**0.53–0.74**)	**0.94** (**0.90–0.98**)	**0.67** (**0.57–0.79**)	**0.84** (**0.76–0.92**)	0.84 (0.64–1.1)
Secondary I complete	1.02 (0.92–1.13)	**0.61** (**0.58–0.63**)	NA	**0.58** (**0.53–0.63**)	NA	**0.53** (**0.45–0.62**)	NA	**0.46** (**0.34–0.63**)	**0.76** (**0.73–0.8**)	NA	NA	**0.62** (**0.43–0.90**)
Secondary II complete	**1.37** (**1.10–1.70**)	0.73 (0.68–0.78)	0.93 (0.87–0.99)	**0.71** (**0.65–0.78**)	NA	**0.51** (**0.42–0.61**)	NA	**0.29** (**0.24–0.37**)	**0.72** (**0.68–0.76**)	**0.45** (**0.36–0.56**)	**0.62** (**0.56–0.68**)	**0.43** (**0.29–0.65**)
Tertiary complete	**1.66** (**1.46–1.88**)	0.59 (0.56–0.62)	0.71 (0.64–0.80)	**0.69** (**0.63–0.75**)	NA	**0.35** (**0.29–0.41**)	NA	**0.26** (**0.19–0.35**)	**0.66** (**0.63–0.7**)	**0.38** (**0.28–0.51**)	**0.48** (**0.43–0.55**)	1.05 (0.72–1.54)

*Note:* Statistically significant values are bold.

Abbreviation: NA, not available.

People who live in rural areas of Brazil, Colombia, Ecuador, El Salvador and Mexico are more likely to die at home with OR ranging from 1.1 (95% CI = 1.03–1.18) in Colombia to 3.11 (95% CI = 2.61–3.7) in El Salvador.

In all countries, people dying of hematologic cancer were more likely to die in hospitals than those with solid tumors. The OR ranged from 0.24 (95% CI = 0.2–0.29) in Guatemala to 0.52 (95% CI = 0.44–0.63) in Uruguay.

People with higher levels of education were less likely to die at home from cancer, with the exception of Argentina, where patients who had completed secondary II and tertiary education were more likely to die at home than those who had not completed primary school (OR = 1.37, 95% CI = 1.10–1.70 and OR = 1.66, 95% CI = 1.46–1.88, respectively).

Results by gender and marital status were inconsistent across the countries. Results from the sensitivity analysis (Tables S2 and S3) supported these findings.

### 3.3 Comparison Between Countries

**FIGURE 2:**
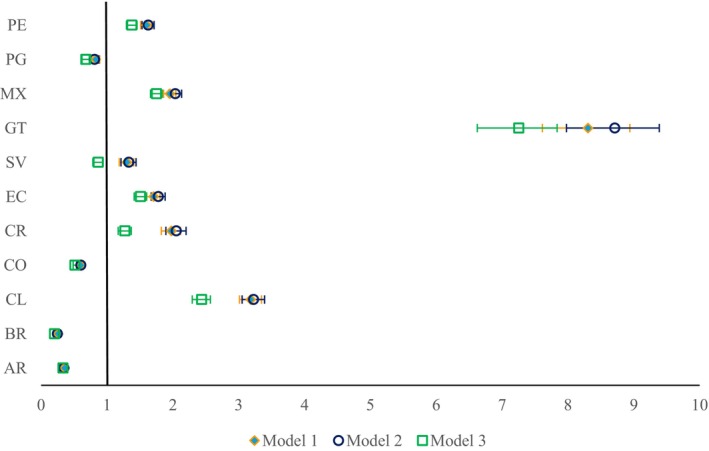
Country differences in the chances of cancer‐related home death versus hospital death. Odds ratio and 95% confidence interval calculated with logistic binary regression with Uruguay as the reference country. Model 1: Country as an independent variable, Model 2: Additionally, type of cancer (solid vs. hematological) as an independent variable, Model 3: Additionally, age, sex, level of education and marital status as independent variables. Comparison of models to evaluate if added variables explain part of the country variations. ORs getting closer to one indicate that by adding variables, part of the variation in the chance of PoD at home to Uruguay can be explained by the variables. A table with numbers can be found in Table S5.

Figure 2 shows the OR of cancer‐related home death versus hospital death in each country compared to Uruguay. ORs close to one indicate that the chance of dying at home in a particular country is similar to the chance in Uruguay. In Model 1, the only independent variable included is the country. In Argentina, Brazil, Colombia and Paraguay, cancer‐related death at home was less likely than in Uruguay, being least likely in Brazil with an OR of 0.26 (95% CI = 0.24–0.27). The chance of dying at home was more likely in Chile, Costa Rica, Ecuador, El Salvador, Guatemala, Mexico and Peru, with an especially high chance in Guatemala, where the likelihood for people with cancer to die at home was eight times higher than in Uruguay.

After adding the cancer type (solid vs. hematologic) as an independent variable in Model 2, the country differences remained largely unchanged. Adding sociodemographic factors in Model 3 showed a slight change in the ORs with reducing differences between Uruguay and half of the countries.

There was a moderate negative correlation between the number of hospital beds, Universal Health Coverage and the percentage of home deaths (*r* = −0.6 and *r* = −0.63). Additionally, the number of physicians, health expenditure per capita and Human Development Index showed a low negative correlation (*r* = −0.47; *r* = −0.39; *r* = −0.31). More people with cancer tend to die in hospitals in countries with more physicians and hospital beds, better coverage of essential health services, more investment in health care, and a higher Human Development Index.

On the other hand, there was negligible correlation between the percentage of home deaths and the number of nurses/midwives and gross national income per capita, Gini Index, distribution of opioids per cancer deaths, the existence of national palliative care law, availability of palliative care services and palliative care development index. Table 3 shows ecological health system‐related factors and correlations to the percentage of home death.

**TABLE 3:** Ecological and health‐care related factors and their correlation with home deaths [21, 22, 23, 24].

Country	Percentage of home deaths^a^	Health expenditure per capita (current US$)	Hospital beds per 10,000 inhabitants	Physicians per 10,000 inhabitants	Nurses and midwives per 10,000 inhabitants	GNI per capita, (current US$)	Palliative care services located in primary care per million inhabitants (2020)	GINI‐index	HDI	UHC	DOME per cancer death (g)	ALCP‐index	National PC law
AR (2017)	19.5	1526	50	40	26	13,140	1.03	41.1	0.85	79	12.16	2.5	No
BR (2017)	15.1	937	21	22	97	8670	0.59	53.3	0.76	82	6.36	5.0	No
CL (2016)	66.8	1180	21	23	113	13,440	12.69	44.4 (2017)	0.85	80.5^b^	18.39	4.4	No
CO (2017)	30.0	495	17	21	13	6010	0.77	49.7	0.76	80	13.05	−1.4	Yes
CR (2016)	56.7	871	12	29	30	11,000	14.13	48.7	0.8	79^b^	8.67	2.6	Yes
EC (2017)	53.5	464	14 (2016)	22	28	5890	2.08	44.7	0.76	78	3.79	2.0	No
SV (2017)	47.4	336	12	22.5^b^	19	3670	0.31	38.0	0.67	78	8.95	3.0	No
GT (2017)	82.0	265	4	3	12	4100	0.23	48.3 (2014)	0.64	58	4.92	−2.8	No
MX (2017)	57.8	515	10	24	29	9310	0.6	47.2^b^	0.78	74	2.65	−8.0	Yes
PY (2017)	35.2	406	8 (2016)	15 (2018)	8	5810	2.9	48.5	0.72	73	NA	NA	No
PE (2017)	51.9	333	16	13.5^b^	22	6030	0.25	43.3	0.77	75	7.14	−3.0	Yes
UY (2018)	38.7	1697	24 (2017)	50	101	17,520	21.9	39.7	0.82	82.5^b^	5.42	75.0	No
Pearson's *R*		−0.39	−0.60	−0.47	−0.12	−0.20	0.12	−0.08	−0.31	−0.63	−0.12	−0.22	0.11

*Note:* GINI‐Index measures the equality of income distribution: 0 = perfectly equal, 100 = imperfect equality; HDI = Human Development Index measures three key dimensions of human development: (1) life expectancy, (2) access to education, (3) decent standard of living (0–1, higher values show better development); UHC = Universal Health Coverage: Index Indicator of the coverage of essential health services by the WHO (0–100, higher values show better coverage); DOME = Distributions of oral morphine equivalents (mean 2015–2017); DOME per cancer death: DOME (mean 2015–2017) divided through number of cancer death from dataset.

Abbreviations: NA, not available; PC, palliative care.

^a^ Age‐adjusted, missing were left out.

^b^ Mean between the year before and the year after.

## 4 Discussion

### 4.1 Main Results

This study provides the first multi‐country comparison of the place of death among individuals who died from cancer across 12 Latin American countries using population‐level data. We observed considerable variations in the proportion of cancer‐related deaths at home (vs. in the hospital), ranging from 14.9% in Brazil to 81% in Guatemala. Across all countries, older age and solid (vs. hematologic) cancers were consistently associated with a higher likelihood of home death. However, individual‐level factors, such as socioeconomic factors or cancer type alone, were insufficient to explain cross‐country differences in place of death. We found that higher healthcare expenditure, better Universal Health Coverage, greater physician density, a higher number of hospital beds, and a higher Human Development Index were all associated with a lower proportion of home deaths.

### 4.2 Interpretation of Cross‐Country Comparison

The proportion of home deaths among cancer decedents varied considerably. The variation exceeded the range reported in the literature (between 11.8% in South Korea [17] and 57.3% in Mexico [12]). As the high variation in the percentage of home deaths in our study could not sufficiently be explained by including sociodemographic factors and cancer type to the model, we considered health system‐related factors as one possible explanation and estimated the correlation between the percentage of home deaths and ecological factors.

When examining health system‐level characteristics, we found consistent associations between higher health care expenditure, more extensive Universal Health Coverage, more physicians and hospital beds, and a higher Human Development Index that tended to have more proportions of hospital deaths. These findings suggest that countries with more developed healthcare systems may deliver a higher proportion of end‐of‐life care within hospital settings. Nevertheless, the modest strength of these associations suggests that health system factors only partially account for the observed variation. This aligns with existing literature, which is also inconclusive about associations between PoD and hospital density [6, 13, 16, 17], and previous studies have found only a small amount of inter‐country variations explained by ecological factors in European countries [5, 12, 17].

Since we were unable to include other possible influencing factors of PoD such as cultural aspects, or details about medical conditions and comorbidity, we did not make assumptions about the reasons underlying the pronounced variation between the countries. However, our study shows that as people with similar diseases and sociodemographic factors die in different places, each country, health system, and community handles end‐of‐life care differently. We found no correlation between the percentage of home deaths and the existence of national palliative care laws and palliative care development. This further indicates variations between health systems in emphasis on palliative care services promotion and the fact that some countries are more hospital‐centric than others.

Furthermore, there was no significant association between the percentage of home deaths and the number of palliative care services located in primary care. Thus, a high proportion of home deaths in Latin American countries does not necessarily indicate better palliative care and adequate end‐of‐life care at home even though most patients report home as the preferred PoD and home death is widely considered a metric of quality of palliative care [3, 10, 25]. In a recent study from Brazil, home death was even seen as an indicator of an insufficient public health system [8].

### 4.3 Interpretation of Associated Sociodemographic Factors

The strongest determinants of home death emerged as age and cancer type. The likelihood of dying at home increased with older age across all studied countries, consistent with previous studies in Latin America [7, 8, 10, 13, 15]. A similar age pattern was also observed in the Czech Republic, France, Italy, South Korea, Spain, the US, Jordan, Singapore and Johannesburg [6, 17, 26, 27]. However, these reports contrast with findings from Belgium, the Netherlands, England, Norway and Wales where older age *decreased* the likelihood of dying at home [6]. Older persons in Latin America might have larger families to take care of them at home or be homebound due to fragility, comorbidity, and limited hospital and nursing home access. The fact that younger people's deaths may be less accepted could lead to families seeking out more aggressive treatment options in hospitals. The contrast with many European countries may be explained by the higher number of care homes in that region [19], unlike in Latin America, where fewer care homes mean more older persons die at home.

The lower rates of home death for patients with hematologic rather than solid cancers corroborate previous findings [5, 6, 7, 8, 12, 13, 19, 20]. Higher symptom burden such as bleeding and risk of complications associated with hematologic cancers, as well as intensive therapies, often require inpatient care. Furthermore, the illness trajectory of hematologic cancers complicates prognosis and delays initiation of conversations about palliative care. Hematologists seem particularly challenged when it comes to initiating palliative care [7, 20]. Prognosis uncertainties may therefore be a factor that hinders home death.

Geographic factors and lack of access to hospitals may explain why people living in rural areas are more likely to die at home. In Latin American countries, hospitals and especially palliative care services are unequally distributed and often concentrated in big cities [24]. People living in remote areas must undertake long, expensive journeys to reach a hospital, a trend observed worldwide [6, 8, 15, 17, 19].

Our study also raises new questions about how social factors influence place of death. In line with previous studies from Mexico and Brazil [7, 8, 13, 15], people with more education were less likely to die at home and more likely to die in hospital. This finding contrasts with European countries where the more highly educated are more likely to die at home [6]. Education level, an indicator of socio‐economic position, reflects the ability to access various economic, cultural, and social resources to navigate a country's specific health system. Therefore, people with a higher educational level have more possibilities to personally decide about their place of death. It may be suspected that those who have the possibility chose the place of death that offers better care. Thus, in Latin American countries, the preferred choice might be in hospital where Palliative Care Services are mainly located. Instead, in European countries, people that can afford home palliative care rather choose to die at home. However, these findings must be treated with caution as many countries were missing data for educational level.

### 4.4 Implications for Research and Policy

Knowing where people with cancer currently die can help policymakers implement the palliative care component of the comprehensive cancer care control strategy, outlined by the WHO and emphasized by the 2017 World Health Assembly resolution 58.22, ‘Cancer Prevention and Control’ [28]. Access to palliative care at end of life is particularly relevant, raising the crucial question of the best way to ensure that patients dying from cancer can choose their preferred place of death and receive the highest quality of care.

Our study suggests that, in the analyzed countries, dying at home may sometimes be the result of a *lack* of access to healthcare, which is largely provided in hospitals. Since palliative care services in Latin America are currently hospital‐centric [24], many patients in rural areas, especially the lower educated and older ones, may be dying without adequate end‐of‐life care. As stated before, the preferred PoD was often reported to be at home, and people in remote areas may be forced to choose between dying in their communities and receiving adequate medical support. Hence, to achieve adequate end‐of‐life care, it is necessary to invest and implement palliative care in primary care and support end‐of‐life care at home, which also reduces costs for the healthcare system [29]. It is also important to enhance palliative care provision within hospitals for patients needing more intensive care. Countries may consider allocating these towards increasing palliative care in primary care settings.

Furthermore, our findings question the assumption that a high proportion of home deaths necessarily indicates high quality of palliative care within a country or adequate end‐of‐life care at home, even though many patients identify home as the preferred PoD [30]. In the region, while some home deaths may represent the desired place of death, and part occur due to insufficient access to appropriate support and essential access to medical services [30].

For a comprehensive understanding of the current state, future research should assess the conditions under which patients currently die and the integration of palliative care in hospitals and home‐based settings. Additionally, it should analyze the preferences and needs of patients, family members, and caregivers, and focus on regional differences and disparities within the country.

### 4.5 Strength and Limitations

To our knowledge, our study is the first to compare cancer‐related PoD between Latin American countries, thus concentrating on a region of diverse middle‐income countries and enabling comparison with existing studies in higher‐income countries. Using integrated death certificate‐based mortality databases from different countries allowed us to study PoD on a population level, thereby limiting selection bias. However, the use of death certificate data has some limitations. Previous studies found that register systems in low‐income and middle‐income countries (LMICs) can be incomplete [31] and inaccurate, including errors in the underlying cause of death or other variables. Furthermore, information on sociodemographic characteristics was sometimes missing. Marital status was unrecorded in 13.10% of cases in Uruguay, 9.2% in Peru and 9.1% in Colombia. The highest percentage of missing data for educational level was 71.2% in Uruguay, 67.3% in Argentina, 23.4% in Peru, 18.3% in Brazil, 16% in Colombia and 15% in Paraguay. We included these cases to reduce the impact of data quality and provide a total population analysis. As reported before by Seitz et al. [18], the quality and feasibility assessment found data from El Salvador and Peru to be low quality, while the remaining countries were rated as medium to high quality. In general, the given data was feasible to conduct our analysis, though higher quality of certificates and more homogeneity between countries could enhance future comparisons.

## 5 Conclusion

This study provides a comprehensive analysis of cancer‐related place of death (PoD) in 12 Latin American countries, highlighting significant cross‐country variations and identifying key factors associated with dying at home versus in a hospital. The study shows that a substantial proportion of cancer deaths occur at home, with notable differences. Older persons, persons with solid cancers, residents of rural areas, and people with lower educational levels are more likely to die at home.

Sociodemographic and ecological factors explain cross‐country variations in the percentage of cancer‐related home deaths to some extent and influence access to hospitals. More hospital beds and higher levels of Universal Health Coverage enabled more patients to access and die in hospitals. However, this only partially explains the variation, suggesting that other factors, such as complex health system‐related and cultural elements, significantly influence how countries handle end‐of‐life care and where patients die. Knowing the current country‐specific distribution of cancer‐related PoD can help stakeholders determine how to enhance palliative care in peoples' actual places of death and better meet their needs.

## Author Contributions

**Alisa Dannenberg:** formal analysis (lead), methodology (lead), project administration (supporting), writing – original draft (lead). **Joachim Cohen:** conceptualization (supporting), methodology (supporting), supervision (supporting), validation (supporting), writing – review and editing (supporting). **Luc Deliens:** conceptualization (supporting), methodology (supporting), supervision (supporting), validation (supporting), writing – review and editing (supporting). **Katja Seitz:** conceptualization (supporting), data curation (lead), methodology (supporting), project administration (supporting), validation (supporting), writing – review and editing (supporting). **Alicia Krikorian:** formal analysis (supporting), writing – review and editing (supporting). **Luis Fernando Rodrigues:** formal analysis (supporting), writing – review and editing (supporting). **Verónica Kramer:** formal analysis (supporting), writing – review and editing (supporting). **Alejandra Sosa Basaistegui:** formal analysis (supporting), writing – review and editing (supporting). **Alberto Alonso Babarro:** formal analysis (supporting), writing – review and editing (supporting). **Andrea Cartin:** formal analysis (supporting), writing – review and editing (supporting). **Celina Castaneda:** formal analysis (supporting), writing – review and editing (supporting). **Lesly Peralta:** formal analysis (supporting), writing – review and editing (supporting). **Cesar Villacres:** formal analysis (supporting), writing – review and editing (supporting). **Sofía Bunge:** formal analysis (supporting), writing – review and editing (supporting). **Tania Pastrana:** conceptualization (lead), formal analysis (supporting), investigation (supporting), methodology (lead), project administration (supporting), software (supporting), supervision (lead), validation (supporting), visualization (supporting), writing – review and editing (supporting).

## Conflicts of Interest

The authors declare no conflicts of interest.

## Supporting information


Data S1.

## Data Availability

The data supporting the findings of this study are available from various national statistical offices. However, some of these data are subject to restrictions, as they were used under license specifically for this study and are not publicly accessible. Data can be made available upon reasonable request from the corresponding author, with the necessary permission from the relevant national statistical office.
