# Supplementary material for: Place of Death of People With Cancer in 12 Latin American Countries: A Population Study Using National Death Registers
Source: Cancer Med. 2025 Jun 18;14(12):e70996. doi: 10.1002/cam4.70996 (PMC12175479; doi:10.1002/cam4.70996)
Supplement: Supplementary file 1 — Data S1. [file CAM4-14-e70996-s001.docx]

**Supplementary data**

**Table S1: Death from cancer and sociodemographic characteristics in 12 Latin American countries, including missing values.**

| **Country** | **total Population** | **Cancer deaths** | **Cancer type %** | **Sex**  **%** | | **Age**  **%** | | | | | **Residence**  **%** | | **Marital status**  **%** | | | | | | **Level of education*****  **%** | | | | | |
| --- | --- | --- | --- | --- | --- | --- | --- | --- | --- | --- | --- | --- | --- | --- | --- | --- | --- | --- | --- | --- | --- | --- | --- | --- |
|  |  |  | hematological | Female | missing | 0-59 | 60-69 | 70-79 | >80 | missing | rural | missing | Single | married | widowed | divorced | stable relationship | missing | I | II | III | IV | V | missing |
| **Argentina (2017)** | 44 044 811 | 62731 | 6·8 | 48·2 | 0·2 | 23·1 | 25·0 | 27·6 | 23·9 | 0·4 | NA | NA | NA | NA | NA | NA | NA | NA | 6·1 | 17 | 6·3 | 0·7 | 2·7 | 67·3 |
| **Brazil (2017)** | 208 504 960 | 217697 | 7·0 | 47·4 | 0 | 30·3 | 25·2 | 24·3 | 20·2 | 0 | 13·5 | 0 | 23·2 | 43 | 19·8 | 7·9 | 3·2 | 2·8 | 36 | 20·6 | 14·3 | 3·9 | 7·1 | 18·2 |
| **Chile (2016)** | 18 038 879 | 26027 | 7·8 | 47·5 | 0 | 20·4 | 22·3 | 28·2 | 29·1 | 0 | 12·4 | 0 | 23·3 | 64·2 | 8·6 | 2·4 | no option | 1·5 | 46·8 | 19·1 | NA | 27·8 | 6·3 | 0 |
| **Colombia (2017)** | 48 351 671 | 42610 | 9·9 | 50·9 | 0 | 29·3 | 22·9 | 25·0 | 22·8 | 0 | 10·1 | 0·2 | 18 | 34·5 | 19·6 | 5·3 | 13·4 | 9·1 | 18·5 | 37·1 | 10·8 | 7·7 | 9·9 | 16 |
| **Costa Rica (2016)** | 4 945 205 | 4857 | 10·8 | 46·7 | 0 | 25·8 | 22·2 | 24·4 | 27·6 | 0 | 21·5 | 0 | 17·6 | 47·6 | 19·2 | 8·1 | 5·7 | 1·8 | NA | NA | NA | NA | NA | 0 |
| **Ecuador (2017)** | 16 696 944 | 11016 | 11·7 | 50·9 | 0 | 29·2 | 19·6 | 24·0 | 27·2 | 0 | 23·0 | 0 | 29·3 | 45·7 | 15·1 | 5·9 | 2·8 | 1·2 | 14·4 | 49·7 | 12·6 | 7·8 | 10·1 | 5·3 |
| **El Salvador (2017)** | 6 266 654 | 3161 | 10·4 | 57·2 | 0 | 35·8 | 21·0 | 22·6 | 20·6 | 0 | 28·7 | 0 | 43·1 | 43·8 | 6·3 | 3 | 1·9 | 2 | NA | NA | NA | NA | NA | NA |
| **Guatemala (2017)** | 16 087 418 | 7738 | 9·6 | 54·9 | 0 | 38·0 | 21·4 | 21·8 | 18·6 | 0·2 | NA | NA | 51·3 | 47·6 | no option ** | | 1 | 0·1 | 43·9 | 33·1 | 4·5 | 10·3 | 4·1 | 4·1 |
| **Mexico (2017)** | 122 839 258 | 84142 | 11·1 | 51·2 | 0 | 34·6 | 22·4 | 23·7 | 19·3 | 0 | 18·6 | 1·1 | 17·3 | 48·5 | 18·9 | 4·3 | 7·9 | 3·1 | 36·4 | 24·6 | 14·6 | 9·4 | 12·3 | 2·9 |
| **Paraguay (2017)** | 6 355 404 | 4329 | 8·8 | 47·1 | 0 | 34·5 | 23·6 | 23·3 | 18·7 | 0 | NA | NA | 38·3 | 45·4 | 10·2 | 1·5 | 2·9 | 1·8 | 30·4 | 32 | NA | 15·4 | 7·2 | 15 |
| **Peru (2017)** | 31 605 486 | 19737 | 10·4 | 53·6 | 0 | 28·4 | 20·2 | 25·6 | 25·8 | 0 | NA | NA | 32 | 43·9 | 9·1 | 1·8 | 4 | 9·2 | 26·7 | 19·8 | NA | 19·3 | 10·7 | 23·4 |
| **Uruguay (2018)** | 3 427 042 | 7884 | 9·0 | 45·5 | 0 | 17·4 | 22·3 | 228·6 | 31·5 | 0·1 | 1·4* | 68·3 | 13·8 | 39·2 | 23·6 | 10·4 | no option | 13·1 | 4·2 | 15 | 3·8 | 3·1 | 2·6 | 71·2 |
| **Total** |  | 491929 | 8·3 | 48·9 | 0 | 29·4 | 23·8 | 24·9 | 21·8 | 0·1 | 11·6 | 1·3 | 19·8 | 39·0 | 15·6 | 5·3 | 4·3 | 3·3 | 29·4 | 22·6 | 11·0 | 6·9 | 7·6 | 20·9 |

NA = not available

**In Guatemala only single, married, and stable relationship as options for marital status

***Level of education: I = less than primary complete; II = Primary completed; III = Secondary I completed; IV = Secondary II completed; V = Tertiary completed

**Sensitivity Analysis**

We conducted a sensitivity analysis with two additional binary logistic regression models, changing the outcome variable PoD to ‘home vs. elsewhere’ and ‘hospital vs. elsewhere’ to check our findings. PoD labelled as ‘missing’ (1·3%) were excluded, leading to 485,502 cases included in both models. We again included the independent variables age, sex, marital status, type of cancer (hematological vs. solid), level of education, rural or urban residence for each country individually. Variables were checked for multicollinearity with variable inflation factor (VIF) and tolerance tests, and no variable had to be excluded due to collinearity.

**Table S2: Multivariable binary logistic regression per country: associations with home death vs· elsewhere; Odds Ratio and 95% Confidence Interval given·**

| **country** | | **AR** | | **BR** | | **CL** | | **CO** | | **CR** | | **EC** | | **SV** | | **GT** | | **MX** | | **PY** | | **PE** | | **UY** |
| --- | --- | --- | --- | --- | --- | --- | --- | --- | --- | --- | --- | --- | --- | --- | --- | --- | --- | --- | --- | --- | --- | --- | --- | --- |
| **Number included** | | 62023 | | 217654 | | 26027 | | 42592 | | 4848 | | 11016 | | 3161 | | 7673 | | 83300 | | 4328 | | 14999 | | 7881 |
| **Sex (Ref: male)** | | | | | | | | | | | | | | | | | | | | | | | | |
| **female** | | 1·02 (0·98 to 1·06) | | 0·85 (0·83 to 0·87) | | 1·14 (1·08 to 1·20) | | 0·99 (0·95 to 1·03) | | 0·98 (0·87 to 1·0) | | 1·06 (0·98 to 1·15) | | 1·14 (0·97 to 1·33) | | 1·10 (0·97 to 1·25) | | 1·05 (1·02 to 1·09) | | 0·844 (0·74 to 0·97) | | 0·97 (0·91 to 1·04) | | 1·18 (1·07 to 1·31) |
| **Age (Ref: 0 to 59)** | | | | | | | | | | | | | | | | | | | | | | | | |
| **60 to 69** | | 1·24 (1·16 to 1·32) | | 1·19 (1·14 to 1·23) | | 1·17 (1·08 to 1·26) | | 1·15 (1·08 to 1·23) | | 1·02 (0·86 to 1·21) | | 1·09 (0·97 to 1·22) | | 1·51 (1·23 to 1·85) | | 1·08 (0·92 to 1·27) | | 1·27 (1·22 to 1·32) | | 1·15 (0·96 to 1·38) | | 0·95 (0·86 to 1·05) | | 1·22 (1·04 to 1·43) |
| **70 to 79** | | 1·64 (1·54 to 1·74) | | 1·52 (1·47 to 1·58) | | 1·40 (1·29 to 1·51) | | 1·33 (1·25 to 1·42) | | 1·14 (0·96 to 1·35) | | 1·15 (1·03 to 1·29) | | 1·78 (1·45 to 2·18) | | 1·39 (1·17 to 1·64) | | 1·63 (1·56 to 1·69) | | 1·26 (1·05 to 1·51) | | 1·13 (1·03 to 1·25) | | 1·61 (1·38 to 1·87) |
| **80 and older** | | 2·38 (2·24 to 2·52) | | 2·26 (2·18 to 2·35) | | 1·87 (1·73 to 2·03) | | 1·87 (1·75 to 1·99) | | 1·63 (1·37 to 1·95) | | 1·62 (1·44 to 1·82) | | 3·11 (2·51 to 3·86) | | 2·07 (1·68 to 2·54) | | 2·44 (2·32 to 2·56) | | 1·73 (1·42 to 2·11) | | 1·27 (1·15 to 1·40) | | 2·16 (1·85 to 2·53) |
| **Residence (Ref: urban)** | | | | | | | | | | | | | | | | | | | | | | | | |
| **rural** | | NA | | 2·41 (2·34 to 2·49) | | 0·97 (0·90 to 1·05) | | 1·09 (1·02 to 1·17) | | 1·02 (0·89 to 1·18) | | 1·31 (1·19 to 1·44) | | 2·89 (2·44 to 3·41) | | NA | | 2·01 (1·93 to 2·09) | | NA | | NA | | 0·70 (0·45 to 1·08) |
| **Marital Status (Ref: Single)** | | | | | | | | | | | | | | | | | | | | | | | | |
| **married** | | NA | | 0·88 (0·85 to 0·91) | | 1·21 (1·14 to 1·29) | | 0·99 (0·93 to 1·05) | | 1·39 (1·18 to 1·64) | | 1·09 (0·99 to 1·20) | | 0·91 (0·77 to 1·07) | | 1·09 (0·96 to 1·24) | | 0·93 (0·90 to 0·97) | | 1·03 (0·89 to 1·19) | | 1·11 (1·02 to 1·20) | | 1·20 (1·03 to 1·40) |
| **widowed** | | NA | | 0·85 (0·85 to 0·93) | | 1·18 (1·06 to 1·31) | | 0·99 (0·92 to 1·07) | | 1·32 (1·07 to 1·63) | | 1·15 (1·01 to 1·32) | | 0·81 (0·59 to 1·12) | | NA | | 0·91 (0·86 to 0·95) | | 1·16 (0·92 to 1·48) | | 1·21 (1·06 to 1·37) | | 1·13 (0·95 to 1·34) |
| **divorced** | | NA | | 0·82 (0·78 to 0·87) | | 1·10 (0·92 to 1·31) | | 0·93 (0·84 to 1·04) | | 1·15 (0·90 to 1·47) | | 0·96 (0·80 to 1·14) | | 1·05 (0·68 to 1·63) | | NA | | 0·83 (0·77 to 0·89) | | 1·77 (1·04 to 3·02) | | 1·25 (0·98 to 1·59) | | 0·92 (0·76 to 1·12) |
| **stable relationship** | | NA | | 1·24 (1·16 to 1·32) | | NA | | 0·86 (0·79 to 0·93) | | 0·70 (0·53 to 0·93) | | 1·42 (1·11 zo 1·82) | | 1·05 (0·61 to 1·80) | | 2·09 (0·88 to 4·97) | | 1·10 (1·03 to 1·17) | | 1·97 (1·35 to 2·87) | | 2·33 (1·94 to 2·80) | | NA |
| **Cancer type (Ref: solid)** | | | | | | | | | | | | | | | | | | | | | | | | |
| **hematological** | | 0·44 (0·40 to 0·50) | | 0·40 (0·37 to 0·43) | | 0·36 (0·33 to 0·40) | | 0·35 (0·32 to 0·38) | | 0·42 (0·35 to 0·51) | | 0·31 (0·27 to 0·35) | | 0·27 (0·20 to 0·37) | | 0·24 (0·20 to 0·28) | | 0·35 (0·33 to 0·37) | | 0·35 (0·27 to 0·47) | | 0·36 (0·31 to 0·40) | | 0·52 (0·44 to 0·62) |
| **Level of Education (Ref: less than primary)** | | | | | | | | | | | | | | | | | | | | | | | | |
| **Primary complete** | 0·97 (0·89 to 1·05) | | 0·78 (0·75 to 0·80) | | 1·00 (0·92 to 1·07) | | 0·86 (0·81 to 0·91) | | NA | | 0·70 (0·62 to 0·79) | | NA | | 0·53 (0·45 to 0·62) | | 0·92 (0·88 to 0·96) | | 0·65 (0·56 To 0·77) | | 0·84 (0·76 to 0·92) | | 0·83 (0·64 to 1·09) | |
| **Secondary I complete** | 1·02 (0·92 to 1·13) | | 0·62 (0·60 to 0·65) | | NA | | 0·58 (0·54 to 0·64) | | NA | | 0·52 (0·44 to 0·61) | | NA | | 0·36 (0·27 to 0·47) | | 0·75 (0·71 to 0·78) | | NA | | NA | | 0·62 (0·43 to 0·89) | |
| **Secondary II complete** | 1·39 (1·11 to 1·72) | | 0·74 (0·69 to 0·80) | | 0·93 (0·87 to 0·99) | | 0·71 (0·65 to 0·78) | | NA | | 0·49 (0·41 to 0·59) | | NA | | 0·20 (0·17 to 0·25) | | 0·71 (0·67 to 0·75) | | 0·44 (0·35 to 0·54) | | 0·65 (0·57 to 0·69) | | 0·43 (0·28 to 0·65) | |
| **Tertiary complete** | 1·62 (1·43 to 1·83) | | 0·61 (0·58 to 0·65) | | 0·70 (0·63 to 0·78) | | 0·69 (0·64 to 0·75) | | NA | | 0·35 (0·29 to 0·41) | | NA | | 0·17 (0·13 to 0·22) | | 0·65 (0·62 to 0·69) | | 0·37 (0·27 to 0·50) | | 0·48 (0·42 to 0·54) | | 1·03 (0·70 to 1·51) | |

NA= not available

**Table S3: Multivariable binary logistic regression per country: associations with hospital death vs· elsewhere; Odds Ratio and 95% Confidence Interval given**

| **country** | **AR** | **BR** | **CL** | **CO** | **CR** | **EC** | **SV** | **GT** | **MX** | **PY** | **PE** | **UY** |
| --- | --- | --- | --- | --- | --- | --- | --- | --- | --- | --- | --- | --- |
| **number included** | 62023 | 217654 | 26027 | 42592 | 4848 | 11016 | 3161 | 7673 | 83300 | 4328 | 14999 | 7881 |
| **Sex (Ref:male)** | | | | | | | | | | | | |
| **female** | 0·95 (0·91 to 0·99) | 1·20 (1·17 to 1·22) | 0·90 (0·85 to 0·95) | 1·02 (0·98 to 1·07) | 1·07 (0·95 to 1·21) | 0·96 (0·88 to 1·04) | 0·92 (0·78 to 1·07) | 0·98 (0·85 to 1·13) | 0·94 (0·92 to 0·97) | 1·28 (1·13 to 1·46) | 1·04 (0·97 to 1·11) | 0·86 (0·78 to 0·95) |
| **Age (Ref: 0 to 59)** | | | | | | | | | | | | |
| **60 to 69** | 0·79 (0·75 to 0·84) | 0·85 (0·82 to 0·87) | 0·86 (0·79 to 0·93) | 0·88 (0·82 to 0·93) | 0·91 (0·77 to 1·08) | 0·92 (0·82 to 1·03) | 0·64 (0·52 to 0·78) | 0·83 (0·69 to 0·99) | 0·78 (0·75 to 0·81) | 0·99 (0·84 to 1·18) | 1·04 (0·94 to 1·15) | 0·83 (0·71 to 0·97) |
| **70 to 79** | 0·59 (0·56 to 0·63) | 0·70 (0·68 to 0·72) | 0·71 (0·66 to 0·77) | 0·75 (0·70 to 0·80) | 0·83 (0·70 to 0·98) | 0·84 (0·75 to 0·94) | 0·56 (0·45 to 0·68) | 0·65 (0·53 to 0·79) | 0·60 (0·57 to 0·62) | 0·89 (0·75 to 1·06) | 0·85 (0·77 to 0·94) | 0·60 (0·52 to 0·70) |
| **80 and older** | 0·37 (0·35 to 0·39) | 0·50 (0·48 to 0·51) | 0·52 (0·48 to 0·56) | 0·54 (0·50 to 0·57) | 0·54 (0·45 to 0·65) | 0·60 (0·70 to 0·84) | 0·31 (0·25 to 0·38) | 0·40 (0·31 to 0·51) | 0·38 (0·37 to 0·40) | 0·66 (0·54 to 0·79) | 0·75 (0·68 to 0·83) | 0·43 (0·37 to 0·50) |
| **Residence (Ref: urban)** | | | | | | | | | | | | |
| **rural** | NA | 0·51 (0·50 to 0·52) | 1·04 (0·95 to 1·13) | 0·90 (0·84 to 0·96) | 0·99 (0·86 to 1·14) | 0·77 (0·70 to 0·84) | 0·33 (0·28 to 0·39) | NA | 0·49 (0·47 to 0·51) | NA | NA | 1·14 (0·92 to 2·18) |
| **Marital Status (Ref: Single)** | | | | | | | | | | | | |
| **married** | NA | 1·19 (1·15 to 1·22) | 0·85 (0·80 to 0·91) | 1·03 (0·97 to 1·09) | 0·81 (0·69 to 0·96) | 0·90 (0·82 to 0·99) | 1·05 (0·89 to 1·24) | 0·83 (0·72 to 0·95) | 1·14 (1·09 to 1·18) | 1·16 (1·01 to 1·33) | 0·92 (0·85 to 1·00) | 0·92 (0·79 to 1·07) |
| **widowed** | NA | 1·11 (1·07 to 1·15) | 0·88 (0·79 to 0·99) | 1·02 (0·95 to 1·10) | 0·82 (0·66 to 1·01) | 0·87 (0·76 to 1·00) | 1·04 (0·75 to 1·45) | NA | 1·15 (1·09 to 1·21) | 1·05 (0·83 to 1·32) | 0·84 (0·74 to 0·96) | 0·91 (0·77 to 1·08) |
| **divorced** | NA | 1·14 (1·09 to 1·19) | 0·91 (0·77 to 1·09) | 1·08 (0·97 to 1·20) | 0·90 (0·71 to 1·15) | 1·01 (0·85 to 1·21) | 0·87 (0·56 to 1·35) | NA | 1·18 (1·09 to 1·27) | 1·03 (0·61 to 1·73) | 0·81 (0·63 to 1·03) | 1·14 (0·94 to 1·39) |
| **stable relationship** | NA | 0·87 (0·81 to 0·92) | NA | 1·19 (1·10 to 1·29) | 1·51 (1·14 to 2·00) | 0·72 (0·74 to 1·57) | 0·66 (0·38 to 1·15) | 0·42 (0·15 to 1·18) | 0·94 (0·88 to 1·00) | 0·69 (0·48 to 1·00) | 0·42 (0·35 to 0·51) | NA |
| **Cancer type (Ref: solid)** | | | | | | | | | | | | |
| **hematological** | 2·19 (1·99 to 2·41) | 2·39 (2·26 to 2·53) | 2·90 (2·64 to 3·18) | 2·87 (2·62 to 3·15) | 2·45 (2·03 to 2·96) | 3·18 (2·80 to 3·62) | 3·63 (2·73 to 4·81) | 3·40 (2·84 to 4·07) | 3·06 (2·92 to 3·21) | 0·97 (0·78 to 1·20) | 2·82 (2·50 to 3·19) | 1·80 (1·52 to 2·14) |
| **Level of Education (Ref: less than primary)** | | | | | | | | | | | | |
| **Primary complete** | 1·02 (0·94 to 1·10) | 1·26 (1·22 to 1·30) | 1·01 (0·94 to 1·09) | 1·18 (1·11 to 1·25) | NA | 1·41 (1·24 to 1·60) | NA | 1·53 (1·29 to 1·82) | 1·05 (1·01 to 1·09) | 1·35 (1·16 to 1·58) | 1·19 (1·08 to 1·31) | 1·16 (0·89 to 1·51) |
| **Secondary I complete** | 0·99 (0·89 to 1·09) | 1·62 (1·56 to 1·69) | NA | 1·74 (1·60 to 1·89) | NA | 1·83 (1·56 to 2·15) | NA | 1·92 (1·42 to 2·61) | 1·28 (1·22 to 1·34) | NA | NA | 1·57 (1·10 to 2·24) |
| **Secondary II complete** | 0·76 (0·61 to 0·94) | 1·39 (1·30 to 1·48) | 1·07 (1·00 to 1·15) | 1·40 (1·28 to 1·54) | NA | 1·86 (1·55 to 2·23) | NA | 2·65 (2·14 to 3·29) | 1·37 (1·30 to 1·45) | 1·79 (1·47 to 2·19) | 1·60 (1·45 to 1·76) | 2·19 (1·47 to 3·26) |
| **Tertiary complete** | 0·61 (0·54 to 0·69) | 1·87 (1·77 to 1·97) | 1·36 (1·22 to 1·53) | 1·48 (1·35 to 1·61) | NA | 2·76 (2·33 to 3·28) | NA | 2·90 (2·14 to 3·92) | 1·47 (1·40 to 1·54) | 2·14 (1·62 to 2·81) | 1·96 (1·74 to 2·21) | 0·93 (0·64 to 1·35) |

NA= not available

**Cancer type**

In a more detailed analysis concerning the association between cancer type and home death, we conducted another binary logistic regression with the dependent variable being death at home vs. in hospital for each country. We again excluded PoD labeled as ‘missing’ and ‘elsewhere’. The only independent variable included was cancer type, grouped into ‘respiratory’, ‘gastrointestinal’, ‘genitourinary’, ‘hematological’ and ‘other’. As the existing literature showed an association between hematological cancer and hospital death we decided to put hematological cancer as reference.

The results are shown in table 4. In all countries, people with respiratory, gastrointestinal, genitourinary or other cancer showed a higher chance of dying at home than people with hematological cancer. In some of the countries, the ORs differ between the remaining cancer groups. For example, in Guatemala the chance for dying at home was more than 6 times higher for those with gastrointestinal cancer compared to hematological, while it was only 3.56 times higher for those with respiratory cancer, However, these variations are not similar between the countries and there is no group that showed the highest chance of dying at home in all countries. To simplify our discussion and the comparability to existing studies, we decided to put the 4 cancer types into one group and compare ‘solid vs. hematological’ in our main analysis.

**Table S4: Multivariable binary logistic regression per country: associations with home death vs. hospital; Odds Ratio and 95% Confidence Interval given**

| **country** | **AR** | **BR** | **CL** | **CO** | **CR** | **EC** | **SV** | **GT** | **MX** | **PY** | **PE** | **UY** |
| --- | --- | --- | --- | --- | --- | --- | --- | --- | --- | --- | --- | --- |
| **Number included** | **60402** | **207977** | **25444** | **42335** | **4734** | **10812** | **3028** | **7249** | **80884** | **3980** | **14656** | **7709** |
| **cancer type (Ref: hematological)** | | | | | | | | | | | | |
| **respiratory** | **2.12 (1.90 to 2.37)** | **2.68 (2.49 to 2.87)** | **2.34 (2.08 to 2.62)** | **2.90 (2.61 to 3.22)** | **2.49 (1.88 to 3.28)** | **2.71 (2.27 to 3.23)** | **4.06 (2.73 to 6.05)** | **3.56 (2.63 to 4.82)** | **3.00 (2.81 to 3.19)** | **2.46 (1.78 to 3.41)** | **1.89 (1.62 to 2.21)** | **1.48 (1.22 to 1.79)** |
| **gastrointestinal** | **2.46 (2.21 to 2.73)** | **2.58 (2.41 to 2.76)** | **3.54 (3.21 to 3.90)** | **3.30 (3.00 to 3.63)** | **2.55 (2.09 to 3.11)** | **4.00 (3.49 to 4.59)** | **4.29 (3.18 to 5.78)** | **6.43 (5.30 to 7.80)** | **3.58 (3.41 to 3.76)** | **2.27 (1.67 to 3.07)** | **3.53 (3.11 to 4.01)** | **1.79 (1.49 to 2.14)** |
| **genitourinary** | **2.52 (2.26 to 2.80)** | **2.90 (2.71 to 3.10)** | **3.11 (2.80 to 3.45)** | **3.16 (2.87 to 3.49)** | **2.60 (2.10 to 3.22)** | **3.81 (3.30 to 4.39)** | **4.05 (2.98 to 5.52)** | **4.88 (3.96 to 6.02)** | **3.11 (2.96 to 3.27)** | **2.15 (1.60 to 2.91)** | **3.13 (2.74 to 3.57)** | **1.87 (1.56 to 2.25)** |
| **other** | **2.05 (1.83 to 2.30)** | **2.82 (2.63 to 3.03)** | **2.04 (1.81 to 2.3)** | **2.66 (2.40 to 2.96)** | **2.59 (2.05 to 3.26)** | **2.67 (2.27 to 3.14)** | **5.23 (3.80 to 7.20)** | **4.91 (3.82 to 6.30)** | **2.78 (2.63 to 2.95)** | **1..91 (1.37 to 2.67)** | **2.56 (2.21 to 2.96)** | **1.82 (1.48 to 2.23)** |

**Table S5: Country differences in the chances of cancer-related home death vs. hospital death**

(Odds Ratio and 95% Confidence interval) calculated with logistic binary regression with Uruguay as reference country. Model 1: country as independent variable, Model 2: additionally type of cancer (solid vs. hematological) as independent variable, Model 3: additionally, age, sex, level of education, and marital status as independent variables. Comparison of models to evaluate if added variables explain part of the country variations. ORs getting closer to one indicate that by adding variables part of the variation in chance of PoD at home to Uruguay can be explained by the variables

|  | **Model 1** | | **Model 2** | | **Model3** | |
| --- | --- | --- | --- | --- | --- | --- |
| **country** | **OR** | **95% CI** | **OR** | **95% CI** | **OR** | **95% CI** |
| **AR** | 0.36 | 0.34 to 0.38 | 0.35 | 0.33 to 0.36 | 0.33 | 0.32 to 0.35 |
| **BR** | 0.26 | 0.24 to 0.27 | 0.25 | 0.24 to 0.26 | 0.20 | 0.19 to 0.21 |
| **CL** | 3.18 | 3.02 to 3.35 | 3.23 | 3.06 to 3.40 | 2.44 | 2.30 to 2.58 |
| **CO** | 0.60 | 0.57 to 0.63 | 0.60 | 0.57 to 0.63 | 0.51 | 0.49 to 0.54 |
| **CR** | 1.98 | 1.84 to 2.13 | 2.05 | 1.91 to 2.21 | 1.27 | 1.18 to 1.38 |
| **EC** | 1.71 | 1.61 to 1.81 | 1.78 | 1.68 to 1.89 | 1.51 | 1.42 to 1.61 |
| **SV** | 1.30 | 1.20 to 1.42 | 1.33 | 1.22 to 1.45 | 0.87 | 0.79 to 0.95 |
| **GT** | 8.30 | 7.67 to 9.00 | 8.71 | 8.04 to 9.45 | 7.26 | 6.67 to 7.89 |
| **MX** | 1.96 | 1.86 to 2.05 | 2.04 | 1.94 to 2.14 | 1.75 | 1.67 to 1.84 |
| **PG** | 0.83 | 0.77 to 0.90 | 0.81 | 0.75 to 0.88 | 0.68 | 0.63 to 0.74 |
| **PE** | 1.60 | 1.52 to 1.69 | 1.63 | 1.54 to 1.72 | 1.38 | 1.30 to 1.46 |

**Ecological factors: age adjusting**

Instead of calculating with the general percentage of home deaths, we decided to adjust concerning age distribution within a country, as earlier age was seen to be an influence factor on likelihood of cancer-related home deaths. We chose a direct adjustment as described by Hilgers et al. ^1^

$$\sum age group\frac{total number of cancer deaths within age group\times percentage of home deaths per age group within country}{total number of cancer deaths}$$

For example: R*(AR)= $\frac{\left( 144470x14\cdot0\% \right)+\left( 117192x16\cdot9\% \right)+\left( 122603x20\cdot8\% \right)+\left( 107309x27\cdot3\% \right)+(355x11\cdot1\%)}{491929}$=19·3%

**Table S6: Age distribution and age adjusted Percentage of home deaths (R*) per country**

| Country | Age | Cancer Death | PoD at home | Rate | R* |
| --- | --- | --- | --- | --- | --- |
| Argentina |  | | | | 19·3% |
|  | 0-59 | 14492 | 2034 | 14·0% |  |
|  | 60-69 | 15657 | 2649 | 16·9% |  |
|  | 70-79 | 17305 | 3604 | 20·8% |  |
|  | >= 80 | 14997 | 4092 | 27·3% |  |
|  | ignored | 280 | 31 | 11·1% |  |
|  | total | 62731 | 12403 | 19·8% |  |
| Brazil |  | | | | 15·1% |
|  | 0-59 | 66056 | 7018 | 10·6% |  |
|  | 60-69 | 54758 | 6969 | 12·7% |  |
|  | 70-79 | 52839 | 8617 | 16·3% |  |
|  | >= 80 | 44013 | 9907 | 22·5% |  |
|  | ignored | 31 | 2 | 6·5% |  |
|  | total | 217697 | 32513 | 14·9% |  |
| Chile |  | | | | 66·8% |
|  | 0-59 | 5306 | 3180 | 59·9% |  |
|  | 60-69 | 5801 | 3778 | 65·1% |  |
|  | 70-79 | 7343 | 5093 | 69·4% |  |
|  | >= 80 | 7577 | 5697 | 75·2% |  |
|  | ignored | 0 | 0 | 0·0% |  |
|  | total | 26027 | 17748 | 68·2% |  |
| Colombia |  | | | | 30·0% |
|  | 0-59 | 12501 | 2902 | 23·2% |  |
|  | 60-69 | 9768 | 2704 | 27·7% |  |
|  | 70-79 | 10637 | 3358 | 31·6% |  |
|  | >= 80 | 9704 | 3892 | 40·1% |  |
|  | ignored | 0 | 0 | 0·0% |  |
|  | total | 42610 | 12856 | 30·2% |  |
| Costa Rica |  | | | | 56·6% |
|  | 0-59 | 1251 | 638 | 51·0% |  |
|  | 60-69 | 1079 | 585 | 54·2% |  |
|  | 70-79 | 1184 | 676 | 57·1% |  |
|  | >= 80 | 1342 | 889 | 66·2% |  |
|  | ignored | 1 | 0 | 0·0% |  |
|  | total | 4857 | 2788 | 57·4% |  |
| Ecuador |  | | | | 53·5% |
|  | 0-59 | 3214 | 1458 | 45·4% |  |
|  | 60-69 | 2155 | 1105 | 51·3% |  |
|  | 70-79 | 2649 | 1466 | 55·3% |  |
|  | >= 80 | 2998 | 1949 | 65·0% |  |
|  | ignored | 0 | 0 | 0·0% |  |
|  | total | 11016 | 5978 | 54·3% |  |
| El Salvador |  | | | | 47·4% |
|  | 0-59 | 1133 | 406 | 35·8% |  |
|  | 60-69 | 663 | 304 | 45·9% |  |
|  | 70-79 | 713 | 354 | 49·6% |  |
|  | >= 80 | 652 | 406 | 62·3% |  |
|  | ignored | 0 | 0 | 0·0% |  |
|  | total | 3161 | 1470 | 46·5% |  |
| Guatemala |  | | | | 81·4% |
|  | 0-59 | 2942 | 2157 | 73·3% |  |
|  | 60-69 | 1654 | 1321 | 79·9% |  |
|  | 70-79 | 1688 | 1433 | 84·9% |  |
|  | >= 80 | 1439 | 1292 | 89·8% |  |
|  | ignored | 15 | 13 | 86·7% |  |
|  | total | 7738 | 6216 | 80·3% |  |
| Mexico |  | | | | 57·3% |
|  | 0-59 | 29104 | 13492 | 46·4% |  |
|  | 60-69 | 18886 | 10237 | 54·2% |  |
|  | 70-79 | 19929 | 12202 | 61·2% |  |
|  | >= 80 | 16201 | 11478 | 70·8% |  |
|  | ignored | 22 | 3 | 13·6% |  |
|  | total | 84142 | 47412 | 56·3% |  |
| Paraguay |  | | | | 35·2% |
|  | 0-59 | 1492 | 417 | 27·9% |  |
|  | 60-69 | 1020 | 339 | 33·2% |  |
|  | 70-79 | 1007 | 368 | 36·5% |  |
|  | >= 80 | 809 | 368 | 45·5% |  |
|  | ignored | 1 | 1 | 100·0% |  |
|  | total | 4329 | 1493 | 34·5% |  |
| Peru |  | | | | 39·6% |
|  | 0-59 | 5605 | 2075 | 37·0% |  |
|  | 60-69 | 3990 | 1481 | 37·1% |  |
|  | 70-79 | 5052 | 2080 | 41·2% |  |
|  | >= 80 | 5090 | 2237 | 43·9% |  |
|  | ignored | 0 | 0 | 0·0% |  |
|  | total | 19737 | 7873 | 39·9% |  |
| Uruguay |  | | | | 38·7% |
|  | 0-59 | 1374 | 409 | 29·8% |  |
|  | 60-69 | 1761 | 610 | 34·6% |  |
|  | 70-79 | 2257 | 955 | 42·3% |  |
|  | >= 80 | 2487 | 1261 | 50·7% |  |
|  | ignored | 5 | 4 | 80·0% |  |
|  | total | 7884 | 3239 | 41·1% |  |
| Total |  | | | |  |
|  | 0-59 | 144470 | 36186 | 25·0% |  |
|  | 60-69 | 117192 | 32075 | 27·4% |  |
|  | 70-79 | 122603 | 40206 | 32·8% |  |
|  | >= 80 | 107309 | 43468 | 40·5% |  |
|  | ignored | 355 | 54 | 15·2% |  |
|  | total | 491929 | 151989 | 30·9% |  |
|  | | | | | |
|  | | | | | |

**Literature**

1. Hilgers R-D, Bauer P, Scheiber V. Einführung in die medizinische Statistik Berlin, Gemany; Heidelberg, Germany; New York, US; 2007: 261-73.
